# Protective Effects of *Glycine soja* Leaf and Stem Extract against Chondrocyte Inflammation and Osteoarthritis

**DOI:** 10.3390/ijms24054829

**Published:** 2023-03-02

**Authors:** Yun Mi Lee, Eunjung Son, Seung-Hyung Kim, Dong-Seon Kim

**Affiliations:** 1 KM Science Research Division, Korea Institute of Oriental Medicine, Daejeon 34054, Republic of Korea; 2 Institute of Traditional Medicine and Bioscience, Daejeon University, Daejeon 34520, Republic of Korea

**Keywords:** *Glycine soja*, leaf and stem extract, inflammation, chondrocyte, osteoarthritis

## Abstract

Wild soybean, also known as *Glycine soja* Sieb. et Zucc. (GS), has long been known for its various health benefits. Although various pharmacological effects of *G. soja* have been studied, the effects of GS leaf and stem (GSLS) on osteoarthritis (OA) have not been evaluated. Here, we examined the anti-inflammatory effects of GSLS in interleukin-1β (IL-1β)-stimulated SW1353 human chondrocytes. GSLS inhibited the expression of inflammatory cytokines and matrix metalloproteinases and ameliorated the degradation of collagen type II in IL-1β-stimulated chondrocytes. Furthermore, GSLS played a protective role in chondrocytes by inhibiting the activation of NF-κB. In addition, our in vivo study demonstrated that GSLS ameliorated pain and reversed cartilage degeneration in joints by inhibiting inflammatory responses in a monosodium iodoacetate (MIA)-induced OA rat model. GSLS remarkably reduced the MIA-induced OA symptoms, such as joint pain, and decreased the serum levels of proinflammatory mediators, cytokines, and matrix metalloproteinases (MMPs). Our findings show that GSLS exerts anti-osteoarthritic effects and reduces pain and cartilage degeneration by downregulating inflammation, suggesting that it is a useful therapeutic candidate for OA.

## 1. Introduction

Osteoarthritis (OA) is a chronic progressive degenerative joint disorder characterized by cartilage degradation and physical disability [1]. The development and progression of OA lead to cartilage matrix degradation caused by inflammation, which results in the degradation of the extracellular matrix (ECM) [2]. Many proinflammatory cytokines participate in OA pathogenesis, with interleukin 1 beta (IL-1β), IL-6, and tumor necrosis factor-alpha (TNF-α) being the most important proinflammatory mediators [3,4]. IL-1β is a key inducer of inflammation and directly participates in the generation of multiple inflammatory mediators such as nitric oxide (NO) and prostaglandin E2 (PGE2) [5]. Additionally, cartilage-degrading enzymes such as matrix metalloproteinases (MMPs), which are upregulated by IL-1β in chondrocytes, degrade collagen type II (COL-II) and accelerate the decomposition of the ECM during OA progression [6]. Thus, candidate drugs capable of targeting IL-1β-induced inflammation may be effective as novel therapeutic strategies for OA.

Natural products have been evaluated for their potential in the prevention and treatment of OA, providing an effective and safe adjunctive therapeutic approach. Many studies have investigated herbal medicines and natural products for their suppressive effects on chondrocytes apoptosis, induction of ECM degradation, and prevention of ECM decomposition in articular cartilage [7,8]. *Glycine soja* Sieb. et Zucc. (GS), wild soybean regarded as the progenitor of cultivated soybean has a long history of use in China dating back more than 2000 years; it is considered an excellent source of soybean-derived drugs [9]. GS exhibits various clinically relevant effects, including improvement in blood lipid profile and reduction in hepatic steatosis and adipocyte size in high-fat diet mice [10]. The health benefits associated with polyphenols in soybean are attributed to phenolic acids, flavonoids, and anthocyanins [11,12]. In addition, according to records of folk remedies in an ancient Chinese book, Material Medical for Famines, aerial parts of the plant have proven clinical effects, such as controlling excessive sweating and tonifying the kidneys and spleen [13]. Flavonoids and triterpenoids from the aerial parts of GS exert growth-inhibitory effects against insect pests [14]. However, to date, no studies have reported the therapeutic effects of GS leaf and stem (GSLS), particularly in OA. Therefore, in this study, we investigated the effects of GSLS against inflammation and ECM degradation through the downregulation of MMPs (MMP-1, MMP-3, and MMP-13) via activation of the NF-κB signaling pathway in IL-1β-treated SW1353 cells. We further investigated the potential of GSLS in preventing inflammatory responses and protecting against articular cartilage degradation in a rat model of monosodium iodoacetate (MIA)-induced OA. Our findings reveal that GSLS significantly attenuates the levels of inflammatory markers in chondrocytes and reduces pain and cartilage damage in MIA-induced rats.

## 2. Results

### 2.1. Chemical Profiling of GSLS

**Figure 1:**
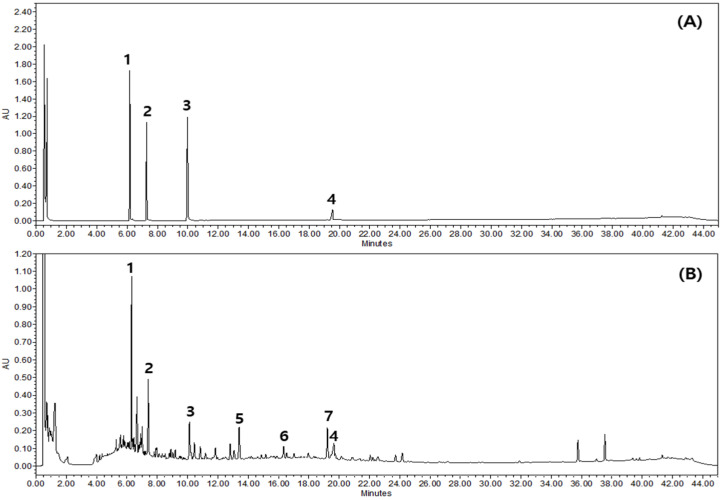
Representative UPLC chromatogram of the 70% ethanolic extract of *Glycine soja* leaf and stem (GSLS). Mixed standard solution (**A**) and 70% ethanol extract of GSLS (**B**). Daidzin (1), genistin (2), daidzein (3), soyasaponin Bb (4), apigenin (5), formononetin (6), and soyasaponin II (7).

Based on the absorption profile and retention time, GSLS contained 3.10 ± 0.008 mg/g daidzin, 2.22 ± 0.039 mg/g genistin, 0.71 ± 0.014 mg/g daidzein, and 7.75 ± 0.343 mg/g soyasaponin Bb. Mass spectroscopy data of GSLS predict the presence of apigenin ([M + H]^+^, 271.12), formononetin ([M + H]^+^, 269.10), and soyasaponin II ([M − H]^−^, 911.46) (Figure 1).

### 2.2. Effects of GSLS on the Viability in Chondrocytes

**Figure 2:**
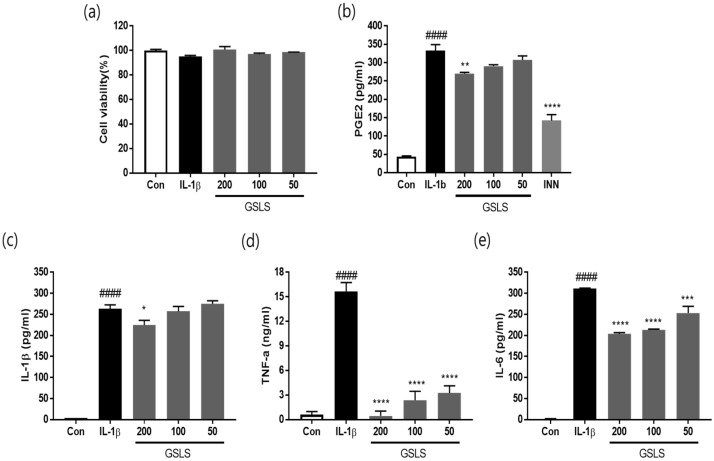
Effects of GSLS on the levels of inflammatory cytokines in interleukin (IL)-1β-stimulated SW1353 chondrocytes. Cells were pretreated with GSLS 2 h before IL-1β stimulation (10 ng/mL) and were maintained for 24 h. (**a**) Cell viability was measured using the MTT assay. The concentrations of PGE2 (**b**), IL-1β (**c**), TNF-α (**d**), and IL-6 (**e**) released into the culture medium were determined using an enzyme-linked immunosorbent assay (ELISA). The values are expressed as the mean ± SD (*n* = 3). ^####^ *p* < 0.0001 vs. untreated control. * *p* < 0.05, ** *p* < 0.01, *** *p* < 0.001, and **** *p* < 0.0001 vs. IL-1β-treated group.

The effect of GSLS on cell viability in SW1353 chondrocytes was examined using the 3-(4,5-dimethylthiazol-2-yl)-2,5-diphenyltetrazolium bromide (MTT) assay. IL-1β and/or GSLS treatment had no significant effect on cell viability after 24 h of incubation (Figure 2a). Therefore, GSLS concentrations at 200 μg/mL or less were used for all subsequent experiments.

### 2.3. Effects of GSLS on IL-1β, TNF-α, and IL-6 Production

The effects of GSLS on the expression of inflammatory factors, IL-1β, TNF-α, IL-6, and PGE2 were analyzed using an enzyme-linked immunosorbent assay (ELISA). As shown in Figure 2b–e, PGE2, IL-1β, TNF-α, and IL-6 levels were significantly increased in the culture medium of the IL-1β-stimulated SW1353 cells. In contrast, the expression of PGE2 was decreased when the cells were treated with GSLS (200 µg/mL) and indomethacin (INN, 2 ug/ml), a positive control, by 21.76%, and 65.97%, respectively. The IL-1β level was markedly reduced (by 14.98%) in cells treated with GSLS (200 μg/mL) along with IL-1β, compared with those treated with IL-1β alone (Figure 2c). In addition, GSLS treatment significantly decreased TNF-α production in IL-1β-stimulated cells in a dose-dependent manner; at GSLS concentrations of 50, 100, and 200 μg/mL, TNF-α levels were decreased to 79.29%, 84.95%, and 97.09% at concentrations 50, 100, and 200 μg/mL, respectively (Figure 2d). IL-6 was significantly suppressed by 31.47% and 34.71% after GSLS treatment at concentrations of 100 and 200 μg/mL, respectively (Figure 2e).

### 2.4. Effects of GSLS on IL-1β-Induced Extracellular Matrix Degradation

**Figure 3:**
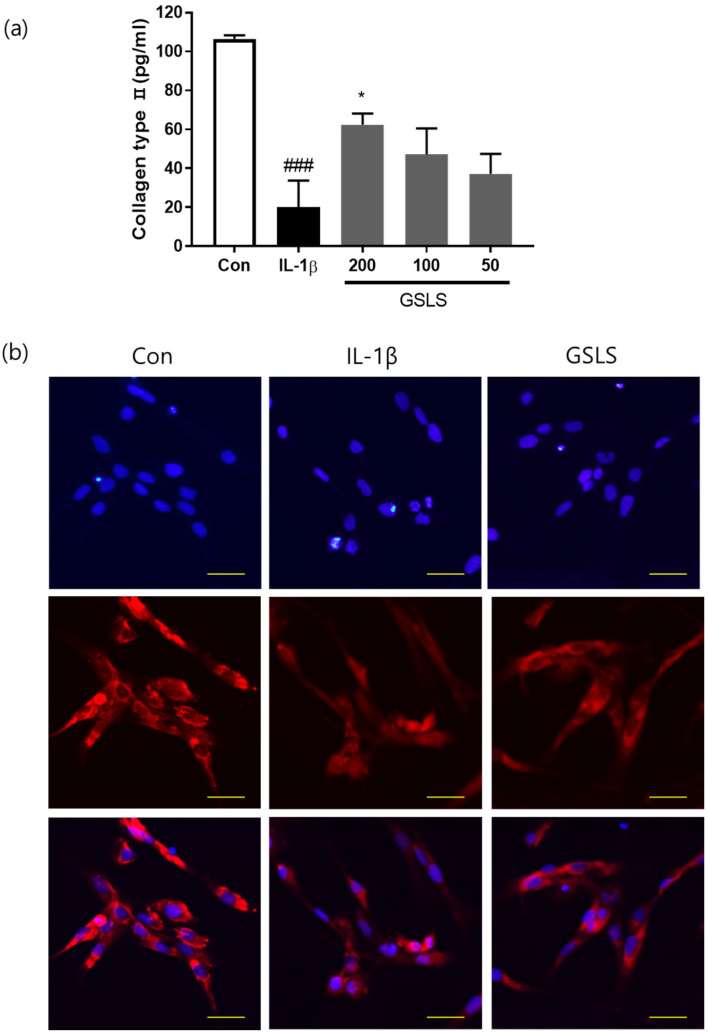
Effects of 200 μg/mL GSLS on IL-1β-induced ECM degradation in IL-1β-stimulated SW1353 chondrocytes. (**a**) Protein levels of COL-II were determined using ELISA. (**b**) Immunofluorescence staining of COL-II protein (red) and nuclei (blue) with 4′,6-diamidino-2-phenylindole (DAPI). Scale bar 20 µm. The values are expressed as the mean ± SD (*n* = 3). ^###^ *p* < 0.001 vs. untreated control. * *p* < 0.05 vs. IL-1β-treated group.

COL-II is the primary component of the ECM and contributes significantly to the support of the cartilaginous structure. ECM degradation during OA is caused by matrix-degrading enzymes such as MMPs [15]. To evaluate chondrocyte degeneration, we investigated the effects of GSLS extract on COL-II levels in IL-1β-treated SW1353 chondrocytes by performing ELISA and immunofluorescence analyses. As shown in Figure 3, COL-II levels were significantly decreased after IL-1β treatment; however, pre-treatment with GSLS at 200 μg/mL prevented this decrease by 49.04%. In addition, immunofluorescence analysis showed that in contrast to the IL-1β group, treatment with GSLS also inhibited IL-1β-stimulated cytoplasmic COL-II degradation.

**Figure 4:**
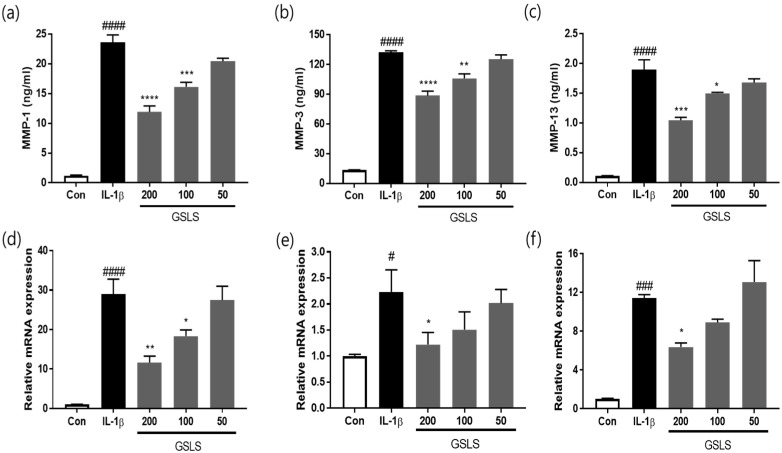
Effects of GSLS on IL-1β-induced expression of matrix metalloproteinase-1 (MMP-1), MMP-3, and MMP-13 in SW1353 chondrocytes. (**a**–**c**) Protein levels of MMP-1, MMP-3, and MMP-13 were determined using ELISA. The mRNA expression levels of (**d**) MMP-1, (**e**) MMP-3, and (**f**) MMP-13 were determined using real-time PCR. The values are expressed as the mean ± SD (*n* = 3). ^#^ *p* < 0.05, ^###^ *p* < 0.001, ^####^ *p* < 0.0001 vs. untreated control. * *p* < 0.05, ** *p* < 0.01, *** *p* < 0.001, and **** *p* < 0.0001 vs. IL-1β-treated group.

**Figure 5:**
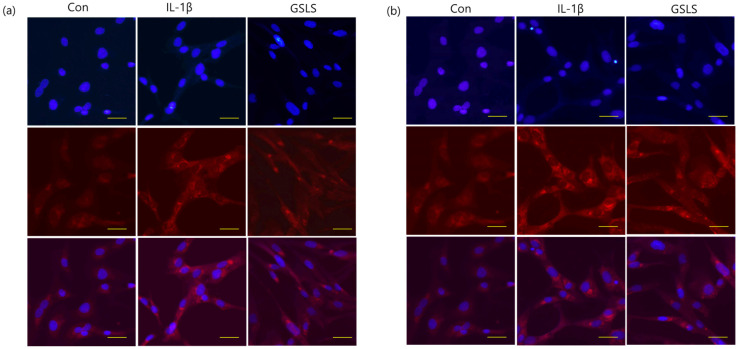
Effects of GSLS on IL-1β-induced ECM degradation using IL-1β-stimulated SW1353 chondrocytes. (**a**) MMP-1 and (**b**) MMP-13 (red) were visualized by immunofluorescence staining. The cells were stained with DAPI to visualize the nuclei (blue). Scale bar 20 µm.

To further investigate how GSLS suppressed IL-1β-induced chondrocyte degeneration, we analyzed the MMP production using ELISA. The results (Figure 4a–c) showed that IL-1β treatment stimulated the protein expression levels of MMP-1, MMP-3, and MMP-13 and increased them to 23.62, 132.35, and 1.90 ng/mL, respectively. However, treatment with 200 μg/mL GSLS decreased these levels to 11.92, 88.72, and 1.05 ng/mL, indicating inhibition of 49.52%, 32.96%, and 44.80%, respectively. In parallel, the effects of GSLS on MMP-1, MMP-3, and MMP-13 mRNA expression were studied using RT–qPCR. As shown in Figure 4d–f, the elevated mRNA expression levels of MMP-1, MMP-3, and MMP-13 were significantly suppressed after GSLS (200 μg/mL) treatment to 59.76%, 45.28%, and 44.13%, respectively. Furthermore, immunofluorescence analysis showed that GSLS protected against IL-1β-induced elevated expression of MMP-1 and MMP-13, which was consistent with the results of mRNA analysis and ELISA (Figure 5a,b). Thus, GSLS exhibits a protective effect on the cartilage matrix by inhibiting ECM degradation via the downregulation of matrix-degrading enzymes and COL-II degradation induced by IL-1β in SW1353 chondrocytes.

### 2.5. Effect of GSLS on IL-1β-Induced NF-κB Signal Activation

**Figure 6:**
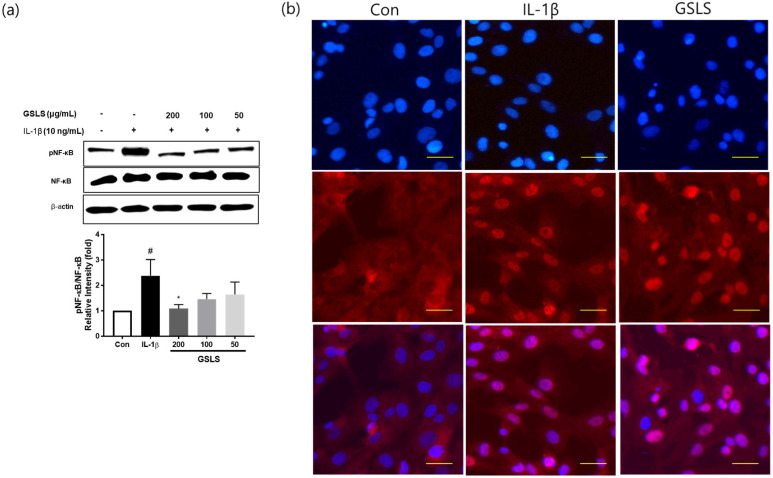
Effects of GSLS on IL-1β-induced NF-κB activation in IL-1β-stimulated SW1353 chondrocytes. Cells were pretreated with GSLS for 2 h, followed by co-incubation with 10 ng/mL IL-1β for 30 min. (**a**) The protein levels of p-p65 and p65 were determined by performing western blotting and quantification analyses. (**b**) The localization of NF-κB p65 was visualized by fluorescence microscopy after immunofluorescence staining (red). The cells were stained with DAPI to visualize the nuclei (blue). Scale bar 20 µm. ^#^ *p* < 0.05 vs. untreated control. * *p* < 0.05 vs. IL-1β-treated group.

To explore the underlying mechanism of the cartilage protective effect of GSLS, western blot and immunofluorescence analyses of NF-κB p65 were performed to examine the effect of GSLS on the NF-κB pathway. IL-1β significantly upregulated p65 phosphorylation (*p* < 0.05), whereas GSLS markedly reduced IL-1β-induced NF-κB activation in a dose-dependent manner (Figure 6a). Furthermore, immunofluorescence analysis revealed that IL-1β-induced nuclear translocation of p65 was significantly blocked by pre-treatment with 200 μg/mL GSLS (Figure 6b). This observation was consistent with the western blotting results. Taken together, these findings demonstrate that GSLS effectively inhibits IL-1β-induced NF-κB signal activation in SW1353 chondrocytes.

### 2.6. Effect of GSLS on Joint Pain in Rats with Monosodium Iodoacetate-Induced Osteoarthritis

**Figure 7:**
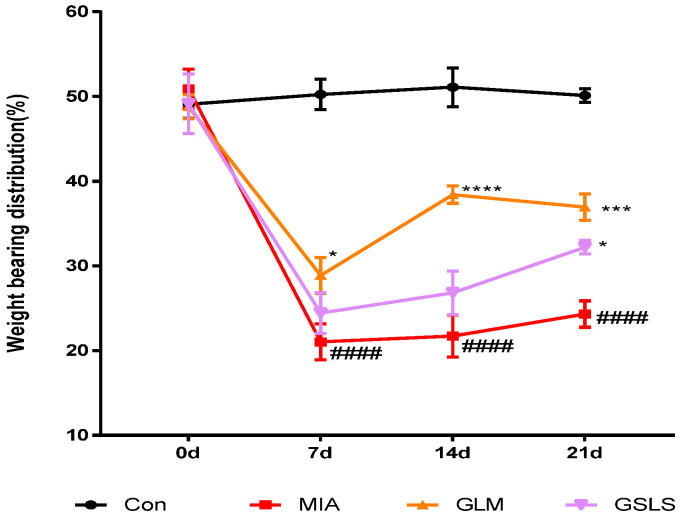
Effects of GSLS on changes in the hind paw weight-bearing distribution in rats with MIA-induced OA. The treatment effects of GSLS were compared with that of the MIA-induced OA group. *n* = 7 per group. ^####^ *p* < 0.0001 vs. control; * *p* < 0.05, *** *p* < 0.001, and **** *p* < 0.0001 vs. MIA-treated rats.

Weight-bearing distribution was measured in sensitized contralateral hind limbs to assess joint pain. We evaluated hind paw weight bearing using an incapacitance tester on days 0, 7, 14, and 21. The weight-bearing in the MIA group decreased rapidly and became significantly different from that of the saline group on day 7 post-MIA injection and was maintained for at least 21 days. These values increased slightly in the GSLS-treated groups on day 7 compared with those in the MIA group. However, in these groups, the balance between the hind legs was restored after 21 days. The 200 mg/kg GSLS and GLM (green-lipped mussel oil) as positive control groups showed maximum pain reduction of 32.4% and 51.9% 21 days post-MIA induction, respectively. These results demonstrated significant recovery of hind paw weight bearing in the GSLS-treated groups (Figure 7).

### 2.7. Effect of GSLS on Inflammatory Mediators and Cytokines in Rats with Monosodium Iodoacetate-Induced Osteoarthritis

**Figure 8:**
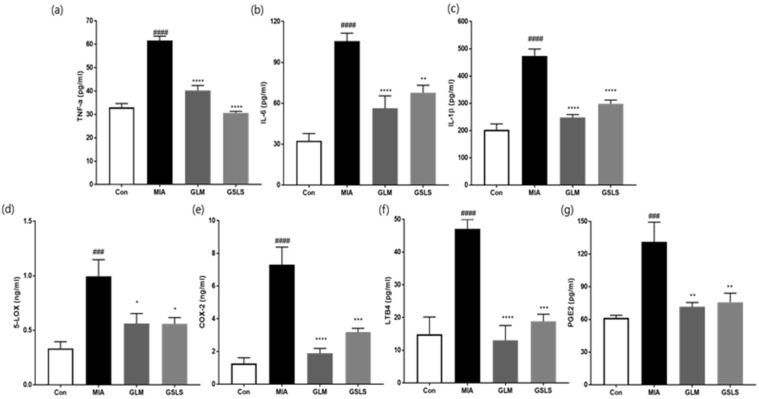
Effects of GSLS on the serum levels of inflammatory cytokines and mediators in rats with MIA-induced OA. (**a**) Serum TNF-α (**b**) IL-6, (**c**) IL-1β, (**d**) 5-lipoxygenase (5-LOX), (**e**) cyclooxygenase-2 (COX-2), (**f**) leukotriene B4 (LTB4), and (**g**) prostaglandin E2 (PGE2) levels. *n* = 7 per group. ^###^ *p* < 0.001, ^####^ *p* < 0.0001 vs. control; * *p* < 0.05, ** *p* < 0.01; *** *p* < 0.001, and **** *p* < 0.0001 vs. MIA-treated rats.

As shown in Figure 8, the levels of inflammatory mediators and cytokines were significantly elevated in the MIA group compared with those in the control group. In contrast, the GSLS-treated group showed remarkably decreased serum levels of IL-1β (64.8%), TNF-α (comparable to levels in control), IL-6 (51.7%), 5-lipoxygenase (5-LOX, 66.0%), cyclooxygenase-2 (COX-2, 68.1%), leukotriene B4 (LTB4, 87.7%), and PGE2 (79.4%) in rats with MIA-induced OA at a dose of 200 mg/kg (Figure 8a–g). These results suggest that GSLS suppresses inflammatory responses by inhibiting the production of inflammatory cytokines and mediators in rats with MIA-induced OA.

### 2.8. Effects of GSLS on Cartilage Degradation in Rats with Monosodium Iodoacetate-Induced Osteoarthritis

**Figure 9:**
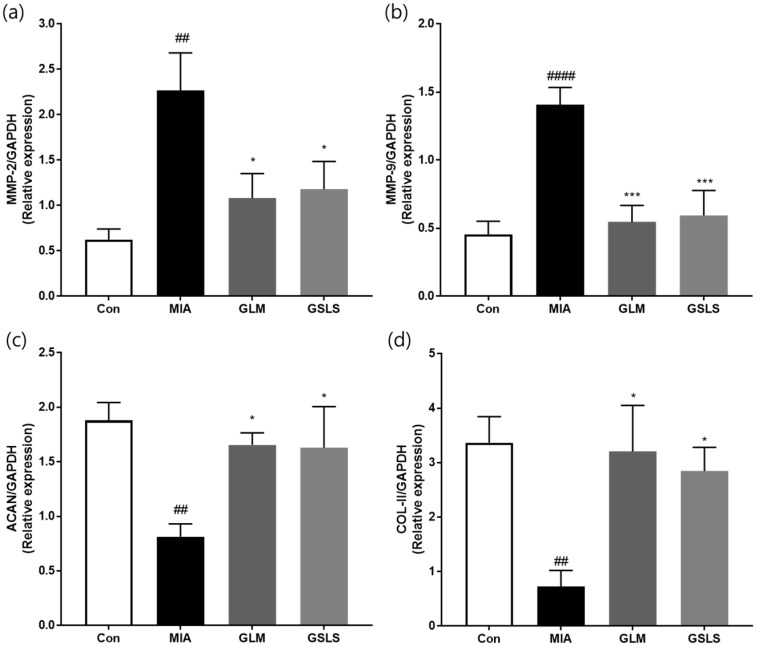
Effects of GSLS on cartilage degradation in rats with MIA-induced OA. The mRNA expression levels of (**a**) MMP-2, (**b**) MMP-9, (**c**) ACAN, and (**d**) COL-II determined by real-time PCR. *n* = 7 per group. ^##^ *p* < 0.01, ^####^ *p* < 0.0001 vs. control; * *p* < 0.05; *** *p* < 0.001 vs. MIA-treated rats.

To assess the inflammatory mediator-promoted secretion of MMPs during the inflammatory process, we next examined whether GSLS inhibited the secretion of MMP-2 and MMP-9 in the knee joint tissues of rats with MIA-induced OA. The MIA group showed elevated mRNA levels of MMP-2 and MMP-9 compared with those in the control group. However, the 200 mg/kg GSLS-treated group showed a significant decrease in MMP-2 and MMP-9 levels (Figure 9a,b). Conversely, mRNA expression of ECM genes such as aggrecan (ACAN), and COL-II, was dramatically reduced in MIA-induced articular cartilage. However, increased ACAN and COL-II mRNA expression levels were observed in the GSLS-treated groups compared with that in the MIA-induced group. These data suggest that GSLS may inhibit cartilage degradation in MIA-induced OA.

## 3. Discussion

OA is a chronic and degenerative joint disease characterized by the destruction of articular cartilage due to an imbalance between biosynthesis and degradation of the ECM, and it leads to physical disability [16]. Although the underlying pathological mechanism of OA remains unclear, inflammatory responses contribute significantly to the symptoms and progression of OA [17]. Various potential natural products and herbal resources have been used to treat OA and/or to delay disease progression [18,19]. The therapeutic effects of GSLS on OA have remained unknown so far. Here, we investigated the effects of GSLS against inflammation and ECM degradation using in vitro and in vivo models and investigated the potential of GSLS in preventing inflammatory responses and protecting against articular cartilage degradation in OA. Our findings reveal that GSLS significantly attenuates the levels of inflammatory markers in chondrocytes and reduces pain and cartilage damage in MIA-induced rats. In previous studies, proinflammatory cytokines, such as TNF-α, IL-1β, IL-6, and IL-10, were detected in synovial fluid from patients with OA and in several experimental animal models of cartilage degradation [20,21,22]. In particular, IL-1β, known as the master regulator of inflammation, has been reported to be directly involved in the generation of multiple inflammatory mediators associated with cartilage degeneration, such as pro-inflammatory factors, PGE2, NO, and MMPs. TNFα is involved in driving the inflammation process [4,5]. Furthermore, the upregulation of IL-1β and TNF-α in articular cells stimulates the production of proinflammatory cytokines such as IL-6 [23] and chemokines such as IL-8 [24]. Hence, in this study, we used IL-1β-stimulated SW1353 cells as an inflammatory chondrocyte model and evaluated the anti-inflammatory effect of GSLS by measuring the changes in the levels of the inflammatory mediators PGE2, IL-1β, IL-6, and TNF-α. Our data revealed that GSLS significantly attenuated these changes in IL-1β-treated chondrocytes. Previous studies have demonstrated that daidzin, genistin, daidzein, and soyasaponin Bb, the main ingredients in GSLS, play roles in the suppression of LPS-stimulated inflammation in macrophages [25,26,27]. Considering these reports of representative compounds, it can be inferred that the therapeutic effects of GSLS could be due to the synergistic anti-inflammatory effects of these compounds. INN, one of the NSAID drugs that reduce the synthesis of prostaglandin by inhibiting cyclooxygenase (COX) to reduce pain, inflammation, and fever, was used as a positive control [28]. INN significantly inhibited the production of PGE2, which was increased by IL-1β in SW1353 cells, but did not show other cytokines inhibitory activities. 

IL-1β induces catabolic responses by suppressing the expression of cartilage-related genes, such as that encoding COL-II [29] and promoting the expression of matrix-degradation-related genes, including those encoding MMP-1, MMP-3, MMP-9, and MMP-13 [30]. The degradation of collagen, one of the ECM components, is an important process in the development, morphogenesis, tissue repair, and remodeling [31]. ECM degradation involves different types of proteases; however, the MMP family members including gelatinases (MMP-2 and -9), collagenases (MMP-1, -8, and -13), stromelysins (MMP-3, -7, -10, and -11), membrane type (MT)-MMPs, and matrilysins are the major enzymes that degrade ECM components [32,33]. In particular, collagenases, including MMP-1, -8, and -13, facilitate the formation of a suitable microenvironment for the development and progression of OA and specifically degrade COL-II and proteoglycans through other MMPs in the cartilage matrix [34]. The activity of gelatinases is higher on the subchondral bone rather than on cartilage ECM [35]. Among MMPs, MMP-3 (also known as stromelysin) activates MMP-1 (also known as collagenase-1) and cleaves a broad range of matrix proteins [36]. MMP-1 is expressed ubiquitously and is found in various normal tissue cells, including chondrocytes, whereas MMP-13 (also known as collagenase-3) is more closely linked to COL-II degradation rather than MMP-1 and MMP-8 and is usually produced only by cartilages and bones during development and by chondrocytes during OA [37,38]. Therefore, the levels of COL-II and MMPs can be used as indicators to investigate the progression of cartilage destruction. In the present study, GSLS inhibited the IL-1β-induced secretion of MMP-1, -3, and -13 in SW1353 chondrocytes. In addition, mRNA and immunofluorescence analyses showed that GSLS treatment reduced the expression of MMP-1 and -13 and increased COL-II in chondrocytes induced by IL-1β. GSLS treatment thus shows a protective effect against ECM degradation and delays OA progression.

The NF-κB pathway mediates important events in the inflammatory response of chondrocytes, leading to progressive ECM damage and cartilage erosion. NF-κB is activated by the inflammatory cytokines IL-1β and TNF-α and mediates the expression of MMPs, including MMP-1, -3, and -13 [34,39,40]. Therefore, NF-κB pathway activation is blocked by drugs currently used for the treatment of OA, such as nonsteroidal anti-inflammatory drugs (NSAIDs) and glucocorticoids, and is a promising strategy for developing novel therapeutics [41]. In our study, GSLS suppressed the signal activation of NF-κB by inhibiting nuclear translocation of p65 induced by IL-1β in chondrocytes. Thus, GSLS has protective effects against inflammation and collagen degradation associated with increased MMPs expression. Considering its anti-inflammatory activity and protective effect against cartilage degradation in vitro, we evaluated the therapeutic potential of GSLS for OA in MIA-induced rats by measuring joint pain, levels of inflammatory cytokines and mediators, and cartilage degradation. GLM, used as a positive control, is produced as a variety of therapeutic supplements and is taken orally as a whole powder or oil extract. GLM is beneficial in relieving pain, reducing inflammation, and ameliorating other debilitating symptoms associated with inflammatory diseases such as OA without the adverse side effects of NSAIDs [42]. The pain-relieving effect of GSLS in MIA-induced rats, as measured by weight-bearing distribution, was markedly higher than that in the MIA-induced group. We observed that GSLS inhibited the production of inflammatory mediators and cytokines, including IL-1β, TNF-α, IL-6, 5-LOX, COX-2, LTB4, and PGE2, in MIA-induced rats. We demonstrated the ECM degradation effect of GSLS by MMP-1, MMP-3, and MMP-13 in SW1353 cells. Furthermore, to confirm the ECM degradation effect by gelatinase (MMP-2, MMP-9) in animal models, we tested the mRNA expression levels of MMP-2, MMP-9, ACAN, and COL-II in animal joint tissues. As shown in Figure 9, treatment with GSLS significantly reduced MMP-2 and MMP-9, while it increased mRNA expression levels of ACAN and COL-II. These results suggest that GSLS blocks gelatinase activity, thereby protecting ECM degradation in MIA-induced OA rats.

Compared to GLM, GSLS has a similar effect and has demonstrated statistical significance in several biomarkers. Considering that the GLM dosage was administered at a higher concentration than that obtained by converting the animal dose to human equivalent doses based on body surface area, the GSLS extract also showed potential as an excellent therapeutic option for OA. Collectively, these results demonstrated that GSLS could ameliorate OA progression by inhibiting the expression of inflammatory factors, relieving pain, and protecting against cartilage damage in MIA-induced rats.

## 4. Materials and Methods

### 4.1. GSLS Preparation

The leaves and stems of GS were collected from the fields of Munkyeong (Chungbuk, Republic of Korea), 100 g of which was extracted with 1.5 L of 70% aqueous ethanol for 3 h under reflux, concentrated under reduced pressure, and freeze-dried. The extraction yield was 12.3%.

### 4.2. Chemical Profiling of GSLS

A Waters Acquity UPLC system equipped with a quaternary pump, auto-sampler, photodiode array detector, and QDa detector with an Acquity UPLC^®^ BEH C18 column (100 × 2.1 mm, 1.7 μm) was used for the analysis (Waters, Milford, MA, USA). Solvent A (water) and solvent B (acetonitrile) were used for gradient elution at a flow rate of 0.5 mL/min, which was carried out as follows: 0–2 min, 5–5% B; 2–5 min, 5–15% B; 5–30 min, 15–55% B; 30–35 min, 55–72% B; 35–40 min, 72–90% B; 40–42 min, 90–100% B; 42–44 min, 100–5% B; and 44–45 min, 5–5% B. The detection wavelength was set at 200 nm. The column temperature was maintained at 40 °C, and the injection volume was 2 µL. A QDa detector equipped with an electrospray ionization (ESI) source and mass spectrometer (MS) was used to obtain mass spectra. The ESI source was analyzed for both negative and positive ions. The optimized ESI source parameters were: capillary voltage 8 kV and probe temperature 600 °C. Nitrogen was used as a curtain, collision, nebulizer, and heating gas.

### 4.3. Cell Culture

SW1353 human chondrocytes were purchased from American Type Culture Collection (ATCC, Manassas, VA, USA) and cultured in Dulbecco’s Modified Eagle’s Medium/Nutrient Mixture F-12 (DMEM/F12), supplemented with 10% heat-inactivated fetal bovine serum (FBS) and 1% penicillin (100 IU/mL) at 5% CO_2_ and 37 °C. The medium was replaced with serum-free DMEM/F12, and 10 ng/mL IL-1β (Sigma-Aldrich Chemical Co., St. Louis, MO, USA) with or without GSLS (50, 100, or 200 µg/mL) was added for an additional 24 h to stimulate the cells. GSLS was dissolved in 100% DMSO, stored at −20 °C, and diluted in PBS immediately before use to obtain a final concentration of 0.1% DMSO. DMSO (0.1%) was used as control (Con).

### 4.4. Cytotoxicity Measurement

The effects of GSLS on chondrocytes were determined using the MTT (Sigma-Aldrich Chemical Co., St. Louis, MO, USA) assay. SW1353 cells were seeded into 96-well plates (5000 cells/well) at 37 °C for 12 h and then treated with GSLS at 37 °C for 1 h before IL-1β (10 ng/mL) treatment at 37 °C for 24 h. In each well, 50 μg MTT solution was added and incubated for 4 h at 37 °C. The supernatant was removed, and the formazan crystals were dissolved in 100 µL dimethyl sulfoxide (DMSO). Absorbance was measured at 570 nm using a microplate reader (Bio-Rad, Hercules, CA, USA) to assess cell viability.

### 4.5. Rat Model of Monosodium Iodoacetate-Induced OA

Male Sprague Dawley (SD) rats (7 weeks old, 190–210 g body weight) were purchased from Orient Bio (Seongnam, Republic of Korea). After acclimation, rats were housed in individual cages and familiarized with the testing procedure. Male Sprague Dawley rats (8 weeks old) were randomly divided into four groups with seven rats in each group: (1) control group, (2) MIA group with MIA injection, (3) GSLS-treated group (200 mg/kg body weight) with MIA injection, and (4) GLM (green-lipped mussel oil, positive control drug)-treated group (100 mg/kg body weight) with MIA injection. MIA solution (3 mg/50 µL in 0.9% saline) was administered as an intra-articular knee injection in the right knee of anesthetized rats under a mixture of ketamine (25 mg/0.5 mL) and xylazine (20 mg/0.2 mL). All drugs were dissolved in 0.5% carboxymethyl cellulose (CMC)-containing saline immediately before use. The rats received 2 mL GSLS or GLM via oral gavage once daily for 21 days after MIA injection until the end of the experiment. Control and MIA group were given the same volume of 0.5% CMC. After GSLS treatment, no evidence of systemic adverse effects was observed in any study group. Changes in the hind paw weight-bearing were determined by the incapacitance tester (Bioseb Co.; Pinellas Park, FL, USA) on days 0, 7, 14, and 21 after intra-articular MIA induction. The hind paw weight-bearing distribution between the right knee joint (with the MIA injection) and the left knee joint (control side) was evaluated as an index of joint pain in the OA joint. All experiments involving animals were approved by the Institutional Animal Care and Use Committee of Daejeon University (DJUARB2022-027, Daejeon, Republic of Korea) and conducted in accordance with the Guide for the Care and Use of Laboratory Animals published by the US National Institutes of Health (Bethesda, MD, USA).

### 4.6. Enzyme-Linked Immunosorbent Assay for Proinflammatory Cytokines and Matrix Metalloproteinases

The levels of proinflammatory cytokines and mediators (IL-1β, TNF-α, IL-6, PGE2, LTB4, 5-LOX, and COX-2), MMPs (MMP-1, MMP-3, and MMP-13), and COL-II were determined using commercial ELISA kits (MyBioSource, San Diego, CA, USA and R&D Systems, Minneapolis, MN, USA), according to the manufacturer’s protocols.

### 4.7. Western Blot Analysis

The cells were lysed using Laemmli sample buffer (Bio-Rad, Hercules, CA, USA), heated at 100 °C for 5 min, and electrophoresed with 30 µg protein/lane on denaturing sodium dodecyl sulfate–polyacrylamide (SDS-PAGE) gel. Proteins were then transferred onto polyvinylidene fluoride membranes (Bio-Rad, Hercules, CA, USA). The protein-blotted membranes were probed with specific targeting primary antibodies (1:1000 dilution, Santa Cruz Biotechnologies, Santa Cruz, CA, USA) for 1 h, washed, and then incubated with horseradish peroxidase-linked secondary antibodies (1:2000 dilution). After 1 h, the membranes were washed three times, and the signals were detected with SuperSignal Chemiluminescence Reagent (cat no. 46640; Thermo Scientific, Atto Corporation, Tokyo, Japan) using an image analyzer (LAS 4000 mini, GE Healthcare Bio-Sciences, NJ, USA).

### 4.8. Immunofluorescence Staining

The cells were stimulated with IL-1β alone or with GSLS and fixed in 4% (*v*/*v*) methanol-free formaldehyde solution (pH 7.4) for 25 min at room temperature. The cells were permeabilized in 0.3% (*w*/*v*) Triton X-100 for 15 min and then blocked in 5% (*w*/*v*) bovine serum albumin for 30 min. Subsequently, the cells were incubated with anti-NF-κB p65 (1:200 dilution, Santa Cruz Biotechnologies, Santa Cruz, CA, USA) and Texas Red-conjugated secondary antibodies (1:100 dilution). The slides were covered with mounting medium containing 4′,6-diamidino-2-phenylindole (DAPI; Vector Laboratories Inc., Burlingame, CA, USA) and visualized using a Fluoview FV10i confocal microscope (Olympus, Tokyo, Japan).

### 4.9. Total RNA Extraction and Real-Time Quantitative Polymerase Chain Reaction

Total RNA was extracted using the RNeasy Mini Kit (Qiagen Inc., Valencia, CA, USA), according to the manufacturer’s instructions. cDNA was synthesized from total RNA using an iScript cDNA Synthesis Kit (Bio-Rad, Hercules, CA, USA) and amplified with SYBR Green Supermix on an ABI StepOnePlus™ Real-Time PCR System (Applied Biosystems, Foster City, CA, USA) according to following conditions: 30 s of denaturation, followed by 40 cycles at 94 °C for 5 s and 60 °C for 35 s. The relative expression of gene-specific products was analyzed using the comparative Ct (2^−ΔΔCt^) method and normalized to the reference gene glyceraldehyde-3-phosphate dehydrogenase (GAPDH). The sequences of primers constructed were as follows: *GAPDH*: F, 5′-CACCCACTCCTCCACCTTTG-3′ R, 5′-CCACCACCCTGTGCTGTAG-3′; *MMP-1*: F, 5′-GACAGAGATGAAGTCCGGTTT-3′ R, 5′-GCCAAAGGAGCTGTAGATGTC-3′; *MMP-3*: F, 5′-ATTCCATGGAGCCAGGCTTTC-3′ R, 5′-CATTTGGGTCAAACTCCAACTGT-3′; *MMP-13*: F, 5′-AGCCACTTTATGCTTCCTGA-3′ R, 5′-TGGCATCAAGGGATAAGGAAG-3′; *ACAN*: F, 5′-GAAGTGGCGTCCAQAAACCAA-3′ R, 5′-CGTTCCATTCACCCCTCTCA-3′; *COL-II*: F, 5′-GCAACAGCAGGTTCACGTACA-3′ R, 5′-TCGGTACTCGATGATGGTCTTG-3′.

### 4.10. Statistical Analyses

All data were analyzed using Prism 7.0 software (GraphPad Software, Boston, MA, USA). Differences were considered statistically significant at *p* < 0.05. Dunnett’s test was used for multiple comparisons among different groups. Data are represented as the mean ± standard error of the mean (SEM). One-way analysis of variance was used to detect significant differences between the control and treatment groups.

## 5. Conclusions

This study demonstrated that GSLS can inhibit chondrocyte inflammation and have a protective effect on MIA-induced OA. GSLS inhibited chondrocyte inflammation and ameliorated cartilage degeneration by suppressing the NF-κB signal activation in SW1353 cells. In an MIA-induced OA rat model, GSLS administration relieved the pain and reversed cartilage degeneration in joints by inhibiting inflammatory responses. Overall, our findings suggested the potential of GSLS as a therapeutic supplement for relieving inflammation in OA.

## Data Availability

The data are contained within the article.
